# Peptides based on the reactive center loop of *Manduca sexta* serpin-3 block its protease inhibitory function

**DOI:** 10.1038/s41598-020-68316-4

**Published:** 2020-07-13

**Authors:** Miao Li, Daisuke Takahashi, Michael R. Kanost

**Affiliations:** 1 Department of Biochemistry and Molecular Biophysics, Kansas State University, Manhattan, KS 66506 USA; 2 Department of Pharmaceutical Health Care and Sciences, Kyushu University, Fukuoka, 812-8582 Japan

**Keywords:** Biochemistry, Immunology, Innate immunity

## Abstract

One innate immune response in insects is the proteolytic activation of hemolymph prophenoloxidase (proPO), regulated by protease inhibitors called serpins. In the inhibition reaction of serpins, a protease cleaves a peptide bond in a solvent-exposed reactive center loop (RCL) of the serpin, and the serpin undergoes a conformational change, incorporating the amino-terminal segment of the RCL into serpin β-sheet A as a new strand. This results in an irreversible inhibitory complex of the serpin with the protease. We synthesized four peptides with sequences from the hinge region in the RCL of *Manduca sexta* serpin-3 and found they were able to block serpin-3 inhibitory activity, resulting in suppression of inhibitory protease-serpin complex formation. An RCL-derived peptide with the sequence Ser-Val-Ala-Phe-Ser (SVAFS) displayed robust blocking activity against serpin-3. Addition of acetyl-SVAFS-amide to hemolymph led to unregulated proPO activation. Serpin-3 associated with Ac-SVAFS-COO^−^ had an altered circular dichroism spectrum and enhanced thermal resistance to change in secondary structure, indicating that these two molecules formed a binary complex, most likely by insertion of the peptide into β-sheet A. The interference of RCL-derived peptides with serpin activity may lead to new possibilities of “silencing” arthropod serpins with unknown functions for investigation of their physiological roles.

## Introduction

**Figure 1:**
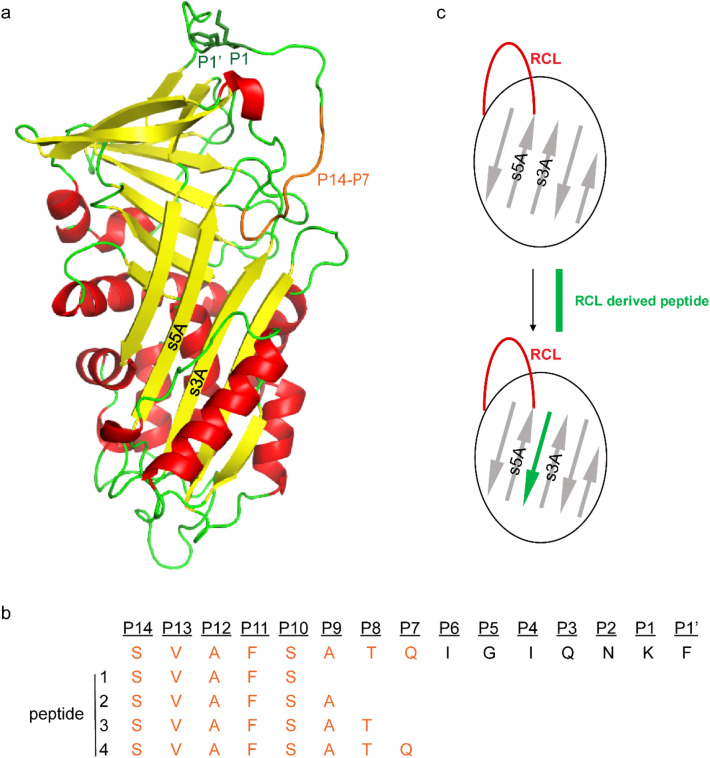
RCL derived peptide design. (**a**) Homology model of serpin-3 made by I-TASSER^46^. P1 and P1′ side chains are indicated as stick drawings and P14-P7 residues are in orange. (**b**) Sequences of RCL derived peptides aligned with P14-P1′ of the serpin-3 RCL. (**c**) A cartoon sketch of native serpin-3 and the complex with RCL-derived peptide inserted into β-sheet A.

Serpins have been extensively studied since the name was coined in 1985 to define a family of serine protease inhibitors with conserved tertiary structure^1^. Broadly distributed throughout vertebrates, invertebrates, plants, unicellular organisms and even viruses, most serpins act as serine protease inhibitors, although some have other functions, including acting as hormone transporters and molecular chaperones^2,3,4,5,6,7^. The tertiary structure of serpins is highly conserved, comprised of three β-sheets, 8–9 α-helices, and a solvent-exposed reactive center loop (RCL) that is near the carboxyl-terminal end of the serpin sequence^2^. When a serpin encounters a target protease, the protease cleaves a specific peptide bond of the RCL (designated P1–P1′, the “scissile bond”) but the second half of the hydrolysis reaction does not occur, leaving the protease and serpin in an acyl-intermediate state. The amino-terminal end of the RCL rapidly inserts into serpin β-sheet A as a new strand (strand 4A) (Fig. 1a), and the protease is translocated to the other side of serpin molecule, resulting in formation of an irreversible inhibitory complex, with the active site of the protease distorted^7^. Alternatively, the cleavage of the scissile bond can proceed as a typical protease hydrolysis reaction, producing a shortened and disabled serpin protein, with no inhibition of the protease. The balance of these two types of serpin-protease interactions determines the efficiency of a serpin as an inhibitor of a given protease^8^.

Rapidly expanding sequence data from insect genomes and transcriptomes has resulted in discovery of thousands of insect serpins, but the physiological functions of only a few have been investigated experimentally through biochemical or genetic studies. Many insect serpins are secreted into the circulating hemolymph and regulate extracellular protease cascades, which activate innate immune responses^3^. By inactivating specific proteases, serpins suppress the immune reactions to avoid excessive production of harmful molecules, which may cause damage to host insects^9^.

Immune responses mediated by hemolymph serine proteases and their regulation by serpins have been studied intensively in a model species, *Manduca sexta*, the tobacco hornworm^10^. The *M. sexta* genome contains 32 serpin genes, and only 10 of these have been studied with regard to their function^11,12^. *M. sexta* serpin-3 expression increases during immune challenge, and it inhibits key proteases in two immune cascades. Serpin-3 inhibits prophenoloxidase activating proteases (PAP), the terminal enzymes in prophenoloxidase activation leading to melanin synthesis, and it inhibits hemolymph protease 8, which cleaves proSpätzle to stimulate the Toll pathway^13,14^. *M. sexta* serpin-3 is orthologous to *Drosophila melanogaster* serpin-27a and *Anopheles gambiae* SRPN2, which are also significant regulators of innate immune protease cascades^15,16^.

Studies of mammalian serpins have shown that peptides with sequences derived from the RCL can modulate serpin function. A synthetic peptide with sequence of the P14-P1 residues of the human α_1_-antitrypsin RCL integrated into α_1_-antitrypsin, and this modified serpin exhibited properties similar to cleaved α_1_-antitrypsin, including lack of inhibitory activity^17^. This finding was followed by studies investigating peptides with sequences derived from the RCL of other human serpins, such as antithrombin, antichymotrypsin, plasminogen activator inhibitor-1, and plasminogen activator inhibitor-2 (PAI-1 and PAI-2)^18,19,20,21^. Characterization of crystal structures of the binary complex of serpin with RCL-derived peptide in antithrombin, PAI-1, and PAI-2 verified the insertion of the RCL-derived peptide as strand 4A in the serpins^21,22,23,24^. In most cases, the principle consequence of RCL-derived peptide incorporation was loss of protease inhibitory activity of the serpin, which acted instead as a substrate of the target protease^25,26,27^. Research in this area has focused on interactions of mammalian serpins and their RCL-derived peptides, because of relevance to serpinopathies, a class of conformational disorders featuring a polymer of serpins in which the RCL of one serpin is inserted into the β-sheet A of an adjacent serpin monomer^28^. There is evidence for potential therapeutic use of RCL-derived peptides to treat serpinopathies, by blocking aberrant polymerization both in vitro and in vivo^29,30^.

These results on mammalian serpins stimulated our investigation to test the use of RCL-derived peptides to manipulate serpin actions in insects, as reagents for experiments to provide a better understanding of serpin physiological functions, particularly for serpins with unknown roles. To test this idea, we hypothesized that serpin-3 from *M. sexta* would be inactivated by synthetic peptides designed based on its RCL sequence, due to binding and insertion of the peptides between β-sheet strands A3 and A5 in place of its own RCL, thus blocking inhibition. We studied a series of peptides with sequences from the serpin-3 RCL and identified an optimum short sequence that blocked inhibition of PAP3 by serpin-3 and also significantly diminished regulation of proPO activation in plasma.

## Materials and methods

### Insects

*M. sexta* eggs originally obtained from Carolina Biological Supply were used to establish a laboratory colony, which has been maintained by feeding on a wheat germ-based artificial diet, with a photoperiod of 16 h of light and 8 h of darkness at 26 °C.

### Synthesis of peptides

Peptides were synthesized using solid phase peptide synthesis on an ABI 431 automated peptide synthesizer (Applied Biosystems, Waltham, MA) with N-Fmoc protected amino acids (P3 Biosystems, Louisville, KY and AnaSpec, Fremont, CA). CLEAR amide resin (Peptides International, Louisville, KY) was used for synthesis of Ac-SVAFSATQ-NH_2_, Ac-SVAFSAT-NH_2_, Ac-SVAFSA-NH_2_, and Ac-SVAFS-NH_2_, while Wang resin pre-loaded with the initial amino acid (AnaSpec, Fremont, CA) was used to synthesize Ac-SVAFS-COO^−^. The amino termini were acetylated using acetic anhydride. The peptides were cleaved from the resin using a solution of 98% trifluoroacetic acid (TFA) and 2% distilled, deionized water. This was mixed at room temperature for 90 min. The slurry was filtered, and the liquid was poured into ice-cold diethyl ether to precipitate peptides. The peptides were then washed with diethyl ether three times and lyophilized. Once dried, the peptides were analyzed using reverse phase HPLC on a Beckman System Gold HPLC machine (Brea, CA) using a C18 column. Solvent A was 99.9% water and 0.1% TFA and Solvent B was 90% acetonitrile, 9.9% water, and 0.1% TFA. A 10% to 90% Solvent B gradient over 30 min was used to elute the peptides. Purity was shown to be over 80%. MALDI-TOF MS was also used for confirmation of the correct mass on a Bruker Ultraflex II machine (Billerica, MA). Lyophilized peptides were dissolved in 2, 2, 2-trifluoroethanol (TFE) before use.

### Expression and purification of PAP3 and serpin-3∆N

Recombinant baculovirus stock for proPAP3 expression was obtained from Dr. Haobo Jiang^31^ and amplified in Sf9 cells in suspension culture in Sf-900 II SFM (Thermo Fisher Scientific) medium. One liter of Sf9 cells (2 × 10^6^ cells/ml) were infected with the recombinant baculovirus at a multiplicity of infection of 2 and cultured at 27 °C with shaking at 140 rpm for 72 h. The medium containing secreted proPAP was harvested by centrifugation at 4 °C, 300×*g* for 10 min. Ammonium sulfate (537 g) was added to the medium, followed by incubation at 4 °C for 2 days without disturbing. A brown precipitate was carefully collected from the surface of medium and then dialyzed against 5 L of 15 mM sodium phosphate, pH 8.0, three times at 4 °C. Immunoblot analysis of this sample using PAP3 polyclonal antiserum (described below) indicated that proPAP3 was cleaved at a position consistent with its proteolytically activated form^32^, due to auto-activation or activation by other proteases in the medium. This sample (~ 150 ml) was then applied to a Ni–NTA column (1.5 × 14 cm, Qiagen), and after washing with buffer (10–30 mM imidazole, 50 mM sodium phosphate, 300 mM NaCl, pH 8.0), PAP3 was eluted with the same buffer supplemented with 250 mM imidazole. The fractions containing PAP3 were dialyzed against 5 L of 20 mM Tris–HCl, 20 mM NaCl, pH 8.0 three times and then concentrated to 3.5 mL by centrifugal ultrafiltration (Amicon 30 K MWCO, Millipore). PAP3 was further purified by anion exchange chromatography on a UnoQ1 column (7 × 35 mm, Bio-Rad) using a linear gradient elution of 20–500 mM NaCl in 20 mM Tris–HCl, pH 8.0. Fractions containing PAP3 were pooled, quantified and stored at − 80 °C.

In a previous study, we found that full length serpin-3 expressed in *E. coli* was not fully active^13^. Therefore, we produced a new construct with deletion of the amino-terminal 30 residues of serpin-3, not present in most other serpins^11^ and found in preliminary experiments that this construct, serpin-3ΔN, retained inhibitory activity. The serpin-3ΔN sequence encoding 31–435 residues of the mature protein was amplified from full-length serpin-3 in vector H6pQE60^13^ by PCR, using a forward primer.

(5′-TACCATGGGCCATCATCATCATCATCACGGCGCAGCTACAGTCACTCCAGAC-3′) that contains an *NcoI* restriction site (underlined) followed by sequence encoding an amino-terminal six-histidine tag (double underlined), and a reverse primer.

(5′-ATGCGGCCGCTCAAGCTTTAAAGGCGCCGTC-3′) that contains a *HindIII* restriction site (underlined). The resulting PCR product was digested with *NcoI* and *HindIII* and purified by agarose gel electrophoresis, then cloned into the same sites in pET28a to produce plasmid pET-serpin-3ΔN, which was used to transfect *E. coli* strain BL21(DE3). Expression of serpin-3ΔN in 1 L *E. coli* culture was induced at mid-log stage by 1 mM IPTG at 37 °C for 6 h. Bacteria were then pelleted by centrifugation and lysed by sonication on ice in 50 mM sodium phosphate, pH 8.0, 300 mM NaCl, 10 mM imidazole. After centrifugation, the pellet and supernatant were subjected to SDS-PAGE, which showed that the majority of serpin-3ΔN was insoluble, but also was present in lower amounts in the soluble fraction. The supernatant (36 mL) was applied to a Ni–NTA (Qiagen) column (1 × 7 cm), and after washing with buffer (10–30 mM imidazole, 50 mM sodium phosphate, 300 mM NaCl, pH 8.0), serpin-3ΔN was eluted with the same buffer supplemented with 250 mM imidazole. Fractions containing serpin-3ΔN were dialyzed against 4 L of 20 mM Tris–HCl, 20 mM NaCl, pH 8.0 three times at 4 °C. Serpin-3ΔN was further purified by chromatography on a UnoQ1 column (7 × 35 mm, Bio-Rad) using a linear gradient of 20–500 mM NaCl in 20 mM Tris–HCl, pH 8.0. Fractions containing serpin-3ΔN were pooled and stored at − 80 °C.

### Protein analysis methods

Protein concentration was measured using Coomassie Plus Protein Assay Reagent (Thermo Scientific), with bovine serum albumin as a standard. Protein samples analyzed by SDS polyacrylamide gel electrophoresis were mixed with 2 × or 6 × SDS loading buffer (supplemented with β-mercaptoethanol) followed by heating at 95 °C for 5 min. Samples were loaded into wells of 4–12% Bis–Tris NuPAGE gels (Invitrogen) and separated by electrophoresis using MOPS buffer. The gels were stained using Instant Blue (Expedeon) or transferred to nitrocellulose in a semi-dry transfer cell (Bio-Rad). The presence of PAP3 or serpin-3ΔN on the membrane was detected using rabbit antisera to PAP3^31^ or serpin-3^13^, followed by goat anti-rabit IgG conjugated to alkaline phosphatase (BioRad) and colorimetric detection of immobilized alkaline phosphatase activity.

### Inhibition of PAP3 by serpin-3 in the presence of RCL-derived peptides

Peptides (25 nmol) in TFE or TFE alone as a control were applied to wells of a 96-well plate and allowed to dry (> 1 h). Serpin-3ΔN (135 ng/7.5 µl) was then added to the wells and incubated at 37 °C for 2 h. PAP3 (108 ng) supplemented with 18 μg bovine serum albumin (BSA) in 46.5 µl 20 mM Tris, 150 mM NaCl, pH 8.0,was then added to the wells and incubated for 10 min at room temperature. The PAP3 substrate N-acetyl-Ile-Glu-Ala-Arg-*p*-nitroanilide (IEAR-*p*NA) at 50 µM in 0.1 M Tris, 0.1 M NaCl, 5 mM CaCl_2_, pH 8.0 was then added, and residual PAP3 activity was measured by the change of absorbance at 405 nm over 20 min at room temperature. Percentage of IEARase activity was defined as IEARase activity divided by IEARase activity of PAP3 in the absence of serpin. One-way ANOVA with Tukey’s multiple comparison as post test was performed in GraphPad Prism. Samples for analysis by reducing SDS-PAGE and immunoblotting were prepared in a similar way. Peptides or TFE were dried in 1.5 mL microcentrifuge tubes and incubated with 7.5 µl serpin-3ΔN (135 ng) at 37 °C for 2 h, followed by addition of 46.5 µl PAP3 (108 ng) supplemented with 18 µg BSA. and further incubated for 10 min at room temperature. Samples were then analyzed by reducing SDS-PAGE and immunoblotting as described above.

To examine the time course of peptide interaction with serpin-3ΔN, pre-dried Ac-SVAFS-NH_2_ or Ac-SVAFS-COO^−^ (400 nmol) were incubated with 40 µL purified serpin-3ΔN (720 ng) supplemented with 40 µL BSA (80 µg) at 37 °C for 1 h. At 0, 10, 20, 40 and 60 min, 12 µL aliquots were withdrawn and immediately incubated with 72 ng of PAP3 at room temperature for 10 min, followed by measuring residual PAP3 activity with 50 mM IEARase in 0.1 M Tris, 0.1 M NaCl, 5 mM CaCl_2_, pH 8.0.

### Effect of RCL-derived peptides on pro-PO activation in plasma

Hemolymph was collected from day 2 fifth instar larvae and centrifuged at 12,000 rpm for 25 min at 4 °C to remove hemocytes, to prepare plasma samples containing the proPO activation system. To examine the effect of pre-incubation of peptide with serpin before exposure to plasma, Ac-SVAFS-COO^−^ (10 nmol in TFE) or TFE alone were dried in wells of a 96-well plate and then incubated with 4 µL serpin-3ΔN (0.1 µg) at 37 °C for 2 h, followed by incubation with 2 μl plasma at room temperature for 10 min. Then the proPO cascade was stimulated by addition of 1 μg *Micrococcus luteus* to activate the proPO protease cascade or filter-sterilized 0.85% NaCl as a control. These mixtures were incubated at room temperature for 10 min, and then PO activity was measured by adding 100 μl of 4 mM dopamine as substrate in 50 mM sodium phosphate, pH 6.5. PO activity was measured by detecting increase in absorbance at 470 nm. One unit of PO activity was defined as 1,000 × ∆A_470_/minute. Three replicates with four different larval plasma samples were examined. Paired one-way ANOVA with Tukey’s multiple comparison as post test was performed in GraphPad Prism.

For an experiment to test the effects of peptide mixed directly to plasma samples, pre-dried Ac-SVAFS-NH_2_ (25 nmol) or TFE in wells of a 96-well plate were incubated with 5 μl plasma for 1 h at room temperature, followed by addition of 95 μl of 50 mM sodium phosphate buffer, pH 6.5 supplemented with or without 2 μg of *M. luteus* (Sigma). After incubation for 10 min at room temperature, PO activity was measured as described above. Three replicates with four different larval plasma samples were examined. Statistical analysis was done as described above.

### Circular dichroism analysis

To examine effects of RCL-derived peptides on serpin-3ΔN secondary structure, 600 µL serpin-3ΔN (150 μg) and 1.8 mL Ac-SVAFS-COO^−^ (25 μmol) were mixed and incubated at 37 °C for 2 h to allow formation of a serpin-peptide binary complex. Then the mixture was subjected to gel permeation chromatography using Superdex 200 10/300 GL (Bio-Rad) in 20 mM Tris–HCl, 150 mM NaCl, pH 8.0 to separate binary complex from free peptide. The fractions containing serpin-3ΔN, presumed to be in complex with peptide, were pooled and quantified. Ellipticity of serpin-3ΔN (0.149 mg/ml) or purified binary complex (0.129 mg/ml) in 10 mM Tris, 5 mM NaCl, pH 8.0 was measured in a 0.01 cm cuvette from 190 to 260 nm at 20 °C. The temperature was then increased in 10 °C increments, and the spectra were measured at each temperature from 20 to 90 °C. The ellipticity of the buffer was also recorded and subtracted from that of serpin-3ΔN and binary complex. The data were smoothed by the method of Savitzky-Golay with value of 15^33^. Mean residue ellipticity was calculated as (ellipticity × mean residue weight)/(10 × 0.01 cm × concentration). Three replicates were examined and results were visualized by GraphPad Prism (means ± standard deviation).

## Results

### Serpin-blocking activities of four serpin-3 RCL-derived peptides

**Figure 2:**
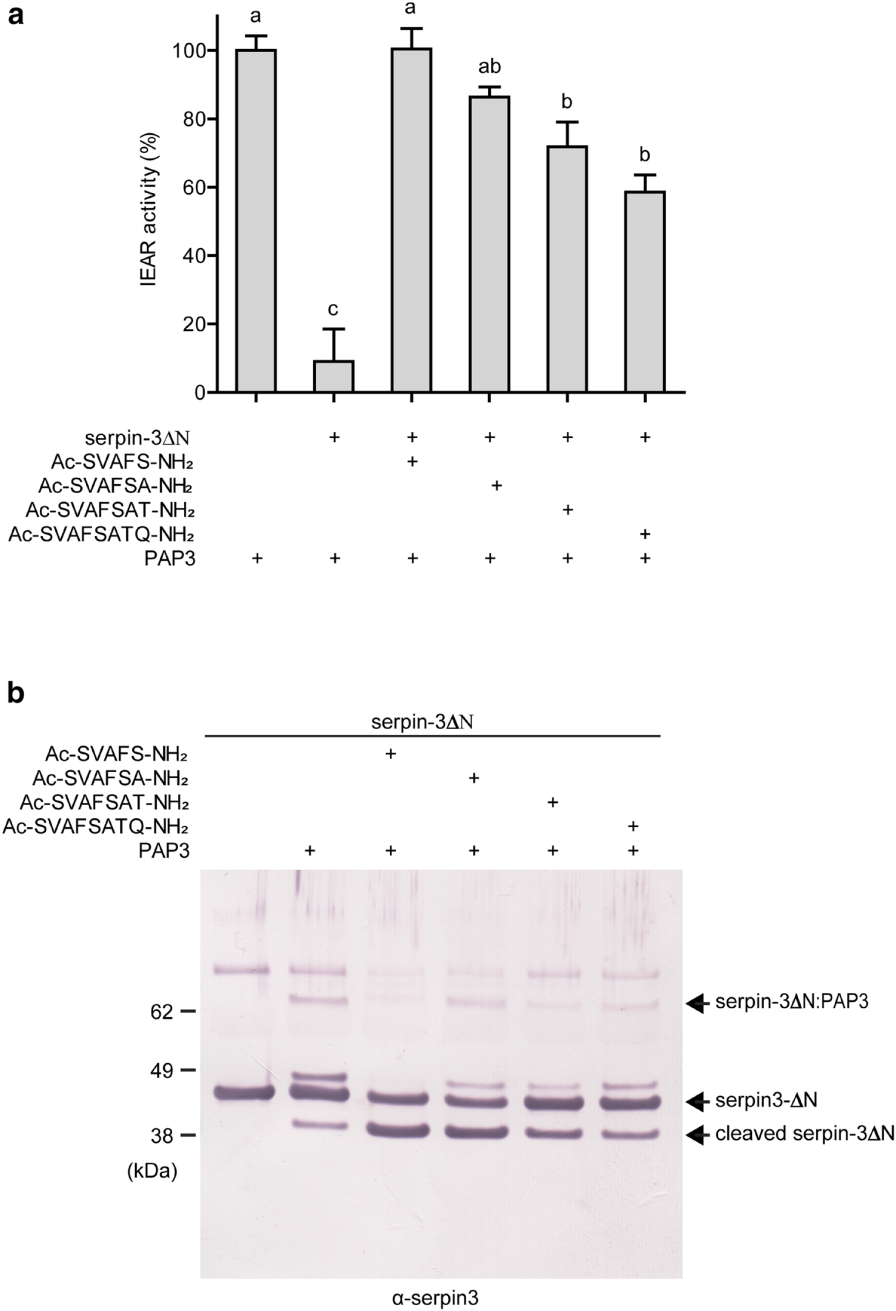
Effects of RCL peptides on inhibitory activity of serpin-3∆N. Pre-dried peptides (25 nmol) or dried TFE as a control were incubated with purified serpin-3∆N (135 ng) at 37 °C for 2 h, followed by addition of PAP3 (108 ng) and further incubation for 10 min at room temperature. (**a**) Residual amidase activitiy of PAP3 was determined by using IEAR-*p*NA (50 µM) as the substrate. Data are means ± standard deviation (n = 3). Results of statistical analysis (one-way ANOVA followed by Tukey’s multiple comparison test, *P* < 0.05) are indicated. Means with the same letter are not significantly different. (**b**) Samples prepared as described above were analyzed by reducing SDS-PAGE followed by immunoblotting using diluted antiserum against serpin-3 as the primary antibody. The identity of a high molecular weight band detected by the serpin-3 antibody is unknown.

We sought to test whether short peptides derived from the serpin-3 RCL would block the inhibitory activity of serpin-3. Based on previous studies of mammalian serpins^17,18,19,20,21,25,26,27^, we designed four peptides that began at the P14 residue of the serpin-3 RCL, with sizes of 5–8 amino acid residues (Fig. 1a, b). These peptides were hypothesized to be incorporated into serpin-3 β-sheet A as a new strand (Fig. 1c). The peptides were initially synthesized with blocking groups at each end (acetyl block at the amino-terminus and amide block at the carboxyl-terminus) to avoid charged groups at the ends of the peptides. The effect of RCL-derived peptides on recombinant serpin-3∆N (Supplementary Fig. S1) was tested by treating the serpin with peptides and then measuring serpin-3∆N inhibition of recombinant PAP3 (Supplementary Fig. S1), one of the main target serine proteases of serpin-3 in regulating *M. sexta* immune responses^13,14^. Serpin-3∆N was an efficient inhibitor of PAP3, with more than 90% inhibition of PAP3 activity (PAP3 activity decreased from 100% ± 4% to 9% ± 9%). Incubation of RCL-derived peptides with serpin-3∆N led to partial loss of inhibitory activity of serpin-3∆N (Fig. 2a). The treatment of serpin-3∆N with the pentapeptide, Ac-SVAFS-NH_2_ (peptide 1, Fig. 1), completely blocked its PAP3 inhibitory activity (PAP3 activity was 100% ± 6%), whereas longer peptides had less effect, in a size-dependent manner. The octapeptide, Ac-SVAFSATQ-NH_2_ (peptide 4, Fig. 1) showed about 50% blockage of serpin-3∆N inhibition of PAP3 (PAP3 activity was 59% ± 5%). The peptides in the absence of serpin had no effect on PAP activity (data not shown).

To further examine molecular effects caused by the RCL-derived peptides, we analyzed samples of serpin-3∆N after reaction with PAP3 in the presence or absence of the four RCL-derived peptides by SDS-PAGE and immunoblotting, using serpin-3 antiserum (Fig. 2b). In the absence of peptides, a major band of intact serpin-3∆N at ~ 42 kDa was present and a covalent inhibitory complex of serpin-3∆N with PAP3 was detected at ~ 75 kDa (Fig. 2b and Supplementary Fig. S2), as previously observed^13^. A minor band at ~ 38 kDa was also present, consistent with some serpin-3∆N undergoing cleavage as a substrate rather than acting as an inhibitor. Another minor band at ~ 48 kDa was only present in the presence of serpin-3 and PAP3, but decreased in intensity when the peptide was present. This band may represent a cleaved form of the serpin-protease complex. In contrast, the incubation of RCL-derived peptides with serpin-3∆N resulted in an enhanced conversion of serpin-3∆N from inhibitor to substrate, indicated by increased intensity of the 38 kDa cleaved serpin band, and decreased formation of the 75 kDa inhibitory complex (Fig. 2b). Consistent with effects on PAP3 amidase activity assay, peptide-1, the shortest RCL-derived peptide, was the most efficient of the four RCL-derived peptides in inducing substrate-like property, with an intense band for the 38 kDa cleaved form, and absence of the ~ 75 kDa complex. Therefore, we used this pentapeptide for further experiments.

### Comparison of effects of Ac-SVAFS-COO^−^ and Ac-SVAFS-NH_2_ on serpin-3

**Figure 3:**
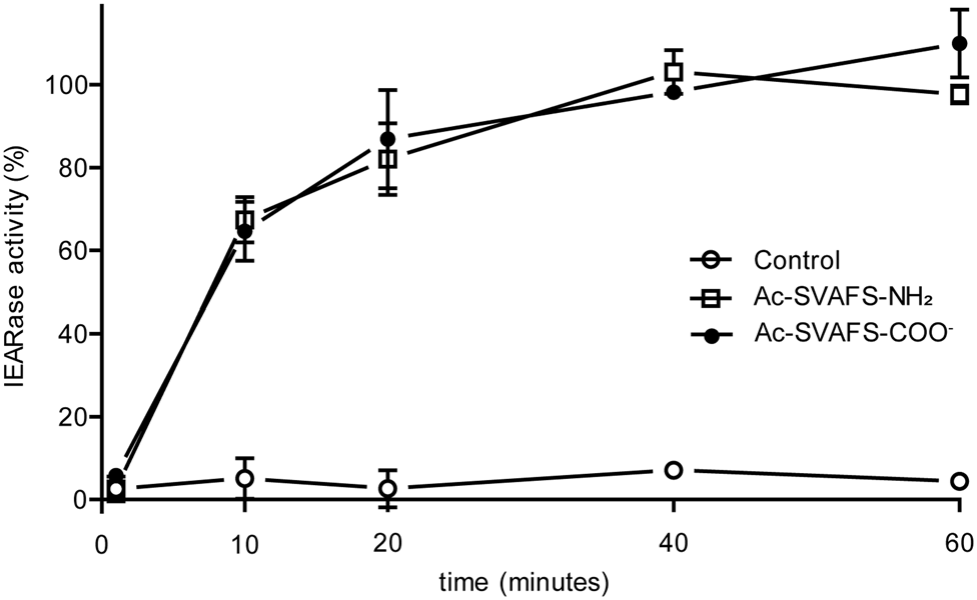
Inactivation of serpin-3∆N by Ac-SVAFS-NH_2_ and Ac-SVAFS-COO^−^ over the time. Pre-dried Ac-SVAFS-NH_2_ and Ac-SVAFS-COO^−^ (400 nmol) were incubated with purified serpin-3∆N (720 ng) at 37 °C for 2 h. A portion of sample was withdrawn at the indicated time intervals and added PAP3 to react at room temperature for 10 min, followed by IEARase activity measurement in 0.1 M Tris, 0.1 M NaCl, 5 mM CaCl_2_, pH 8.0. Percentage of IEARase activity is defined as individual IEARase activity over gross PAP3 activity.

Peptide 1, Ac-SVAFS-NH_2_, with amino-terminal and carboxyl-terminal blocking groups, had very low aqueous solubility. We synthesized a peptide with the same sequence but only an amino-terminal blocking group and a free carboxyl end, Ac-SVAFS-COO^−^, which has a negative charge at neutral pH, for improved solubility. Then we evaluated effects of Ac-SVAFS-NH_2_ and Ac-SVAFS-COO^−^ on the ability of serpin-3∆N to inhibit PAP3 (Fig. 3). Inactivation of serpin-3∆N began to occur by 10 minutes after incubating the serpin with either of the pentapeptides and increased to ~ 100% suppression of serpin-3∆N inhibitory activity by 40 min. This result suggests that the difference in the solubility of these two peptides did not limit their effects on serpin function in this experiment, and that the peptides interact with serpin to cause its inactivation on the scale of minutes.

### Serpin-3 RCL-derived peptide promotes increased PO activation in plasma

**Figure 4:**
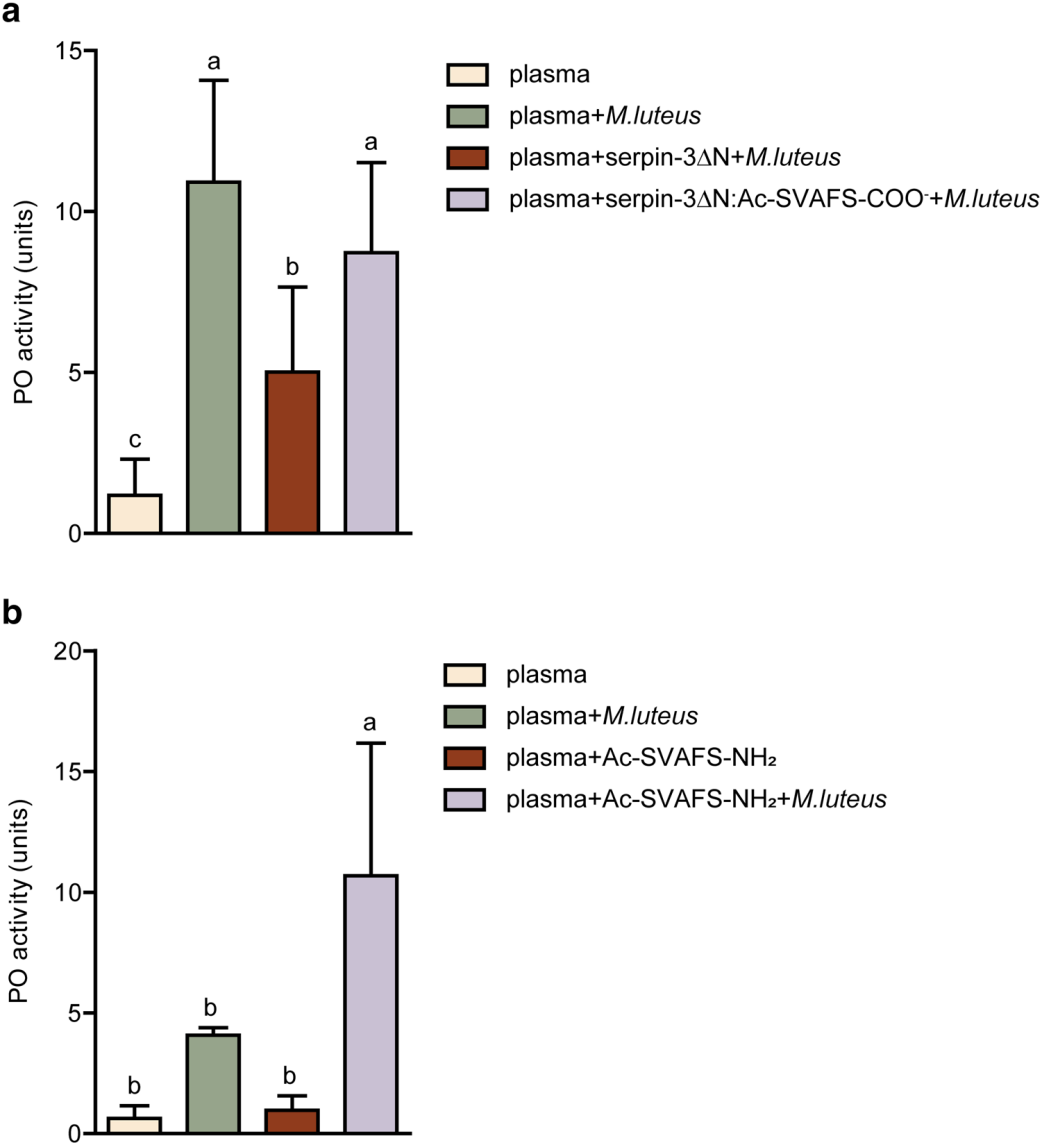
Interference of proPO activation in induced plasma by Ac-SVAFS-NH_2_ and Ac-SVAFS-COO^−^ treated serpin-3∆N. (**a**) Ac-SVAFS-COO^−^ treated serpin-3∆N (0.1 µg) or untreated serpin-3∆N (0.1 µg) was incubated with 2 µl plasma from *M. sexta* larvae at room temperature for 10 min, followed by addition of 2 µg *M. luteus* or sterile saline and further incubation for 10 min at room temperature. PO activities were measured by adding 2 mM dopamine. (**b**) 1 µl plasma from *M. sexta* larvae was incubated with pre-dried Ac-SVAFS-NH_2_ (5 nmol) or TFE in 50 mM sodium phosphate, pH 6.5 at room temperature for 1 h, followed by addition of 2 µg *M. luteus* or sterile saline and further incubation for 10 min at room temperature. PO activities were measured by adding 2 mM dopamine. The graphs represent results using plasma from four different larvae. Individual larva data are shown in Supplementary Fig. S3. One unit of proPO activity is defined as 0.001 change of OD_470_ per minute. Data are means ± standard deviation (n = 4). Results of statistical analysis (paired one-way ANOVA followed by Tukey’s multiple comparison test, *P* < 0.05) are indicated with different letters.

Detection of invading microbes in hemolymph stimulates the initiation of a protease cascade that results in the activation of proPO by PAPs, leading to oxidation of catechols in hemolymph and subsequent reactions that produce melanin^34^. Serpin-3 in hemolymph regulates proPO activation by inhibiting PAPs^13^. To investigate whether the inactivation of serpin-3 by RCL-derived peptides can have an effect in hemolymph, Ac-SVAFS-COO^−^ was pre-incubated with serpin-3∆N to allow formation of the complex, followed by the addition of plasma and stimulation of the protease cascade by *M. luteus* (Fig. 4a and Supplementary Fig. S3). *M. luteus* treatment stimulated activation of PO in plasma (PO activity was 10.9 ± 3.2), which was suppressed by addition of free serpin-3∆N (PO activity was 5.0 ± 2.6). However, addition of serpin-3∆N treated with Ac-SVAFS-COO^−^ to plasma did not significantly attenuate PO activation (PO activity was 8.7 ± 2.8). This result is consistent with lack of inhibition of PAP activity by serpin-3∆N complexed with the peptide.

To further confirm the inactivation of naturally occurring serpin-3 in the complex mixture in hemolymph by RCL-derived peptide, plasma was incubated with Ac-SVAFS-NH_2_ for 1 h to allow the association of RCL-derived peptides with endogenous serpin-3, followed by triggering the proPO cascade by addition of *M. luteus* (Fig. 4b and Supplementary Fig. S3). Addition of the bacteria to plasma led to increased PO activity (from 0.6 ± 0.5 to 4.1 ± 0.3), as expected, but in the presence of Ac-SVAFS-NH_2_, PO activity was significantly greater after addition of bacteria (10.7 ± 5.5) than in control plasma (1.0 ± 0.6) (Fig. 4b), consistent with the hypothesis that the peptide blocked inhibition of PAPs by serpin-3.

### Ac-SVAFS-COO^−^ alters conformation and increases thermal stability of serpin-3∆N

**Figure 5:**
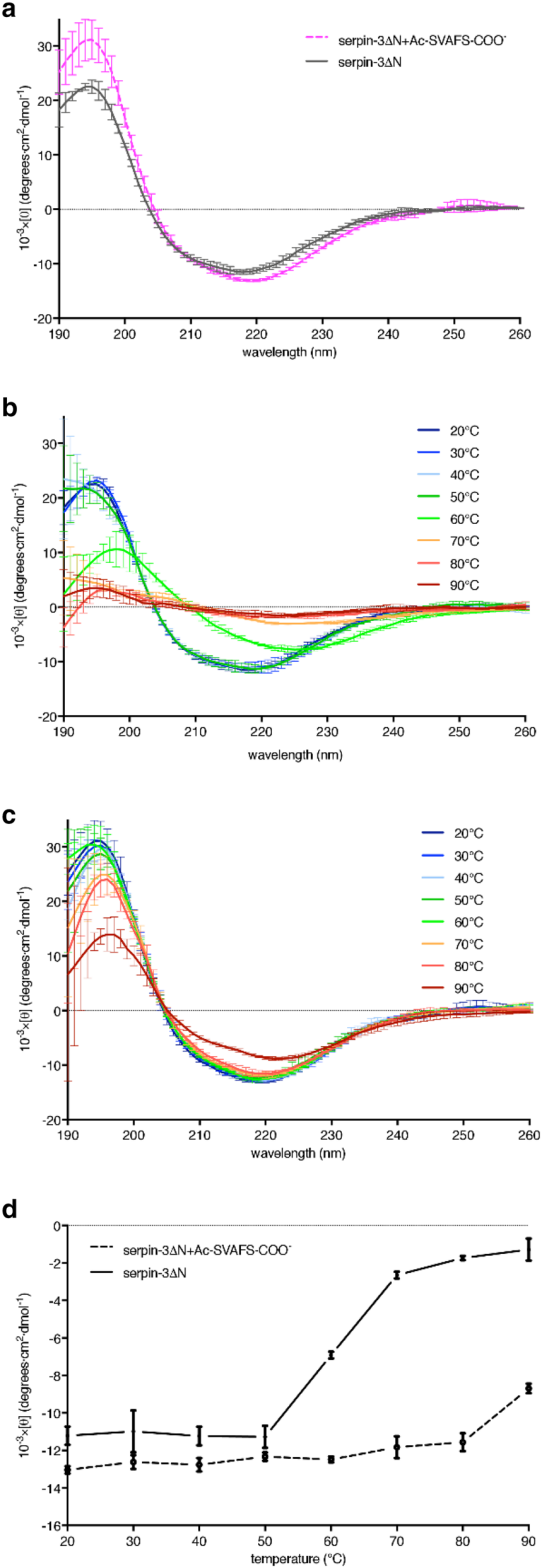
Circular dichroism of serpin-3∆N and binary complex formed by serpin-3∆N and Ac-SVAFS-COO^−^. CD spectra were measured with serpin-3∆N and binary complex formed by serpin-3∆N and Ac-SVAFS-COO^−^ in 10 mM Tris, 5 mM NaCl, pH 8.0 at different temperatures. Data are means ± standard deviation (n = 3). (**a**) CD spectrum of serpin-3∆N and binary complex at 20 °C. (**b**) CD spectrum of serpin-3∆N at different temperatures. (**c**) CD spectrum of binary complex formed by serpin-3∆N and Ac-SVAFS-COO^−^ at different temperatures. (**d**) Mean residue ellipticity of serpin-3∆N and binary complex at 220 nm at different temperatures.

The incorporation of RCL-derived peptides into β-sheet A was confirmed to mimic the relaxed state of human antithrombin, with increased thermodynamic stability^26^. To compare the thermostability of serpin-3∆N with that of the complex formed by serpin-3∆N and RCL-derived peptide, we incubated serpin-3∆N with Ac-SVAFS-COO^−^ and then separated the complex from free peptide by gel permeation chromatography. Analysis of serpin-3∆N and the serpin-3∆N complex by circular dichroism spectroscopy (Fig. 5a) revealed a deeper trough around 220 nm in the complex compared with the free serpin, indicating a slight change in secondary structure in the presence of Ac-SVAFS-COO^−^. We tested the effect of interaction of Ac-SVAFS-COO^−^ on the thermal stability of serpin-3∆N by measuring circular dichroism spectra at increasing temperatures. The spectrum of serpin-3∆N changed significantly at temperatures of 60 °C and above, whereas the serpin-3∆N in complex with Ac-SVAFS-COO^−^ maintained its secondary structure, with little change in CD spectrum up to 90 °C (Fig. 5b and c). Mean residue ellipticity of both native serpin-3∆N and serpin-peptide complex at 220 nm was plotted as a function of temperature (Fig. 5d). The obvious conformational transition of native serpin-3∆N occurred between 50 and 70 °C. In contrast, there was only small and gradual elevation of mean residue ellipticity of the serpin-3∆N complexed with Ac-SVAFS-COO^−^ at higher temperatures, suggesting that the association of serpin with the RCL-derived peptide significantly increased the stability of the serpin molecule.

## Discussion

*M. sexta* serpin-3 regulates proPO activation in response to bacteria by inhibiting PAP-1 and PAP-3 in hemolymph^13^. Complexes formed by serpin-3 with plasma proteases, including PAP-1, PAP-2, PAP-3 and HP8, were identified in plasma by mass spectrometry, confirming the role serpin-3 plays in regulating immune protease cascades in vivo^14^. RNA interference to decrease the expression of a mosquito ortholog of *M. sexta* serpin-3 (SRPN2 in *A. gambiae*) resulted in enhanced melanin deposition on implanted Sephadex beads and the appearance of melanotic pseudotumors on the abdominal wall, indicating that this serpin has a critical role in regulating proPO activation^16,35^. We have not been successful in using RNA interference to decrease expression of plasma proteins in *M. sexta*^36^ and therefore have not been able to determine the effect of lowered serpin-3 concentrations in vivo*.* However, we hypothesized that blocking the inhibitory activity of serpin-3 would result in misregulated proPO activation. Modulation of mammalian serpin function by RCL-derived peptides has been studied through biochemical approaches and showed potential feasibility for application in a mouse model^30^. In this study, we carried out experiments to use regulation of the melanization response by serpin-3 as a model system to investigate the potential for using RCL-derived peptides to probe the functions of insect serpins.

The proximal hinge region in the RCL of serpins tends to have small, hydrophobic residues, allowing this flexible sequence to drive the incorporation of the N-terminal RCL into β sheet-A during protease inhibition, whereas the distal region of the RCL has much more sequence variability in different serpins^37^. Synthetic peptides with sequences spanning P15 or P14 to P1 of the RCL were the first to be described as a counterpart of incorporated strand A4, converting human serpins from an inhibitor to a substrate^17,19,20,21,26^. Furthermore, research with human α_1_-antitrypsin revealed that peptides representing the proximal RCL (P14-P4 and P14-P8) are sufficient to substantially inhibit antitrypsin activity, while those peptides representing the distal part of the RCL (P10-P1, P9-P1 and P8-P1) were much less efficient in blocking α_1_-antitrypsin^38^. Therefore, we designed and synthesized peptides based on the RCL proximal hinge of serpin-3, with length of 5–8 residues (P14-P10, P14-P9, P14-P8 and P14-P7). These peptides did block serpin-3′s ability to inhibit proteolytic activity of PAP3. We found that increasing the length of peptides decreased their efficiency in blocking serpin-3∆N function, with the 5-residue peptide (P14-P10) having the greatest effect (Fig. 2a). This shorter peptide may have easier access for inserting into the space between strand 3A and strand 5A (Fig. 1a). Such a difference was not observed between 11-residue (P14-P4) and 7-residue peptides (P14-P8) derived from the human α_1_-antitrypsin RCL in blocking α_1_-antitrypsin function^38^, perhaps because the longer incubation time they used (24 h) may permit entry of longer peptides into β-sheet A.

In the natural RCL sequence in serpin-3, the amino acid residues from P14 to P2 are not charged, since they are participating in peptide bonds and lack ionizable side chains. In order to mimic the endogenous proximal RCL sequences, we synthesized peptides with ends blocked by N-terminal acetylation and C-terminal amidation, so that they would not contain charged groups at the ends. These peptides blocked serpin-3∆N function, but they had low solubility due to the hydrophobic nature of this segment of the serpin sequence. For this reason, we synthesized the five residue peptide SVAFS with only an N-terminal acetylated group and lacking the C-terminal amidation. This peptide with one negative charge was much more soluble and functioned equally as well as the uncharged peptide (Fig. 3). Therefore, it appears that negative charge at the C-terminus did not impede insertion of the peptide into β-sheet A.

The incorporation of an endogenous RCL into β-sheet A during a protease inhibition reaction is a complex process which requires opening of an existing β-sheet and insertion of a new strand^26^. This involves coordinated movements of the RCL and regions of the serpin called the breach, the shutter, and the gate^39^. Long incubation times (15–48 h) at 37–48 °C are necessary in many cases for inactivation of the human serpins α_1_-antitrypsin, antithrombin, PAI-1 and PAI-2 by corresponding RCL-derived peptides, conditions which probably favor partial unfolding to promote accessibility of the peptides to insert into β-sheet A^17,19,21,22,26,40^. However, we found that the SVAFS peptide can block serpin-3∆N function within 40 min at 37 °C. The relatively small size of this peptide, compared to those investigated in studies of human serpins may contribute to more efficient insertion into β-sheet A. Furthermore, the three-dimensional structure of a mosquito ortholog of serpin-3, *A. gambiae* SRPN2 has the RCL hinge in a “partially inserted” conformation^35,41^, with a few residues from the hinge region inserted into β-sheet A. If *M. sexta* serpin-3 has a similar structure, this might contribute to more efficient insertion of the peptide as a new β-strand in these related insect serpins.

After serpin-3∆N was incubated with the RCL peptides to allow formation of the complex, it lost activity as an inhibitor of PAP, and instead behaved as a substrate for the protease (Fig. 2). This is consistent with observations of human serpins after insertion of RCL-derived peptides into β-sheet A, with increased vulnerability to cleavage in the RCL and lack of inhibitory reactions^20,21,22,23,24,27^.

The CD spectrum of serpin-3∆N complexed with Ac-SVAFS-COO^−^ differed slightly from that of serpin-3∆N alone, with lower ellipticity at 220 nm, similar to observations of human α_1_-antitrypsin and antithrombin in complex with their RCL-derived peptides^17,18,26,38^. The insertion of an RCL or RCL-derived peptide as a new strand (strand A4) in β-sheet A changes the configuration of that region of the β-sheet. Strands 3A and 5A are parallel in the native serpin before insertion of the RCL as strand A4, in a new antiparallel relationship with strands A3 and A5, which increases the stability of overall structure^7^. Native serpins are relatively unstable, with average melting temperature of around 58 °C, while the other forms that contain strand 4A have a much higher melting temperature, such as cleaved serpins with > 110 °C melting temperature^39^, but in some cases the situation is more complex, such as human neuroserpin, which converts from native to very stable latent/polymer form that is not possible to unfold by temperature^42^. Similarly, we found that native serpin-3∆N started to denature at ~ 60 °C, whereas serpin-3∆N in complex with Ac-SVAFS-COO^−^ showed little change in CD spectrum up to the highest temperature 90 °C tested (Fig. 5). This result is quite similar to an experiment with human antithrombin, with change in CD at 220 nm revealing a melting temperature of 58 °C for native antithrombin, but no elliptical change of an antithrombin:P14-P1 complex up to 85 °C^26^. These results support the hypothesis that the RCL peptides are functioning in *M. sexta* serpin-3 in a manner similar to that shown by previous structural and biochemical studies of human serpins.

Because serpin-3 is a negative modulator of the proPO cascade by inhibiting PAPs in hemolymph^13^, we hypothesized that interfering with serpin-3 activity would lead to higher level of proPO activation in response to a microbial challenge. Our results demonstrated that addition of recombinant serpin-3∆N to plasma samples stimulated by *M. luteus* caused a significant reduction in PO activity, likely due to inhibition of PAPs. In contrast, when serpin-3∆N treated with Ac-SVAFS-COO^−^ was added to the same plasma, PO activity was as high as in the absence of added serpin, indicating that the Ac-SVAFS-COO^−^ peptide blocked the inhibition of PAPs by serpin-3∆N, resulting in unregulated proPO activation (Fig. 4). Similar results were observed when we mixed the RCL peptide with plasma, to allow it to interact with endogenous serpin-3 in the complex mixture of hemolymph proteins. Serpin-3 in complex with the peptide did not regulate PAP activity.

RCL peptides have been shown to decrease serpin function in regulating mammalian blood clotting. RCL peptides from plasminogen activator inhibitor-1 (PAI-1) suppress activity of this serpin and result in decreased blood clot lysis *in vitro*^20,27,43^. A series of RCL-derived peptides from mammalian neuroserpin and a myxomavirus serpin displayed anti-inflammatory, anti-atherogenic and pro-thrombotic functions in mice^30^, indicating that RCL peptides can have biological activity in vivo. Another application of RCL-derived peptides is to depolymerize or deaccelerate aberrant polymerization of serpin monomers, as a potential treatment for human serpinopathies^44,45^. Modulating activity of arthropod serpins by RCL-derived peptides may be a useful tool for investigating the biological roles of serpins with unknown functions.

## Supplementary information

Supplementary information

## Data Availability

The authors state that data supporting the findings presented in this article are within the article and/or supplemental materials.
