# Supplementary material for: Peptides based on the reactive center loop of Manduca sexta serpin-3 block its protease inhibitory function
Source: Sci Rep. 2020 Jul 13;10:11497. doi: 10.1038/s41598-020-68316-4 (PMC7359039; doi:10.1038/s41598-020-68316-4)

**Supplementary materials**

**Figure S1**: Purification results of PAP3 and serpin-3ΔN. Purified recombinant PAP3 or serpin-3ΔN with reducing SDS loading buffer was subject to 4-12% Bis-Tris NuPAGE gel. Instant blue staining and immunoblotting using diluted antiserum against PAP3 or serpin-3 as the primary antibody were performed respectively.


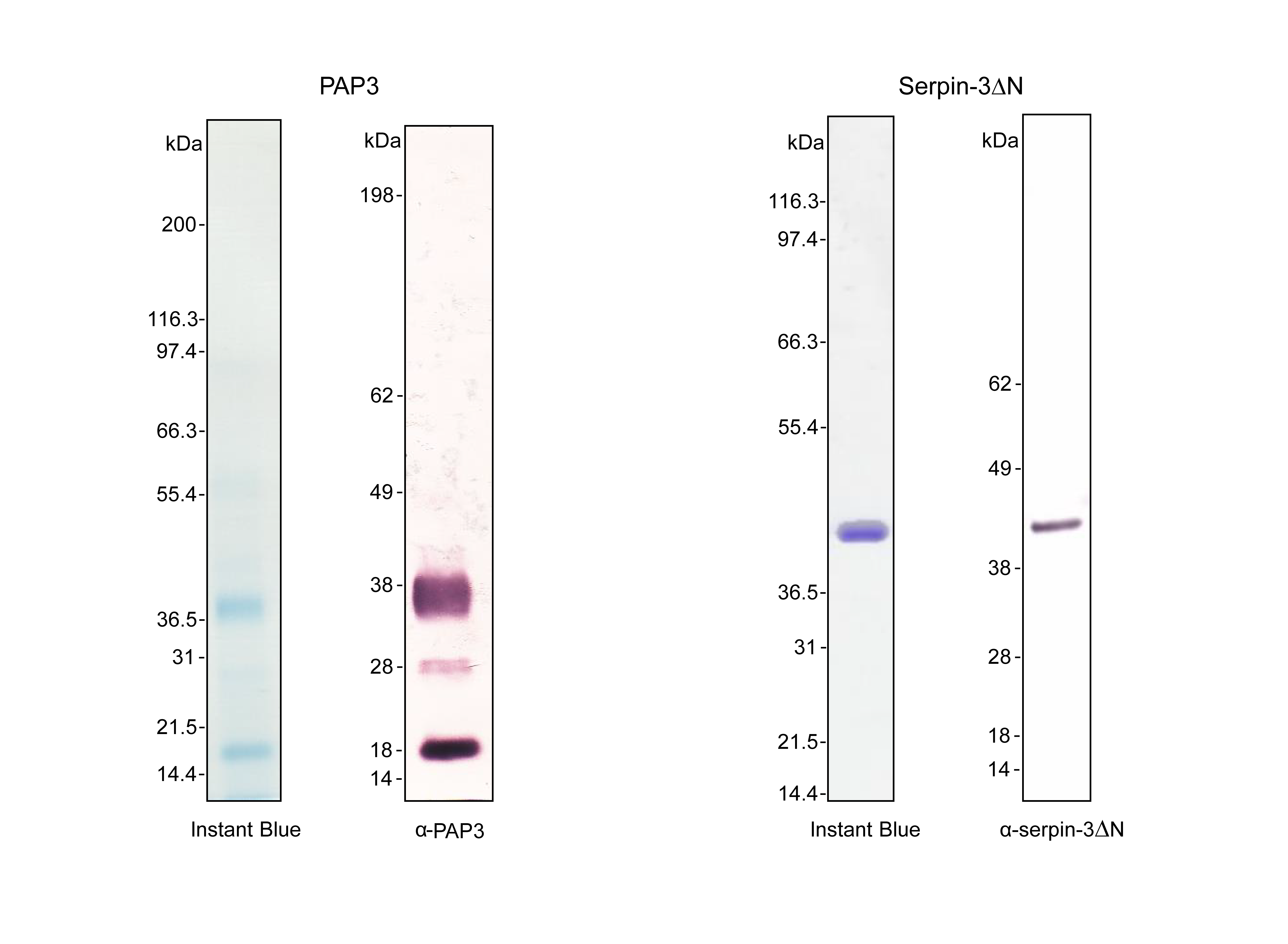


**Figure S2**: Inhibitory complex formed by serpin-3ΔN and PAP3. 0.0014 nmol (72 ng) purified recombinant PAP3 was incubated with 0.0014 nmol (81 ng) or 0.0007nmol (40.5 ng) purified recombinant serpin-3ΔN in 100 mM Tris, pH 8.0 at room temperature for 10 min, followed by analyzing with reducing 4-12% Bis-Tris NuPAGE gel and immunoblotting using diluted antiserum against PAP3 (**a**) or serpin-3 (**b**) as the primary antibody.


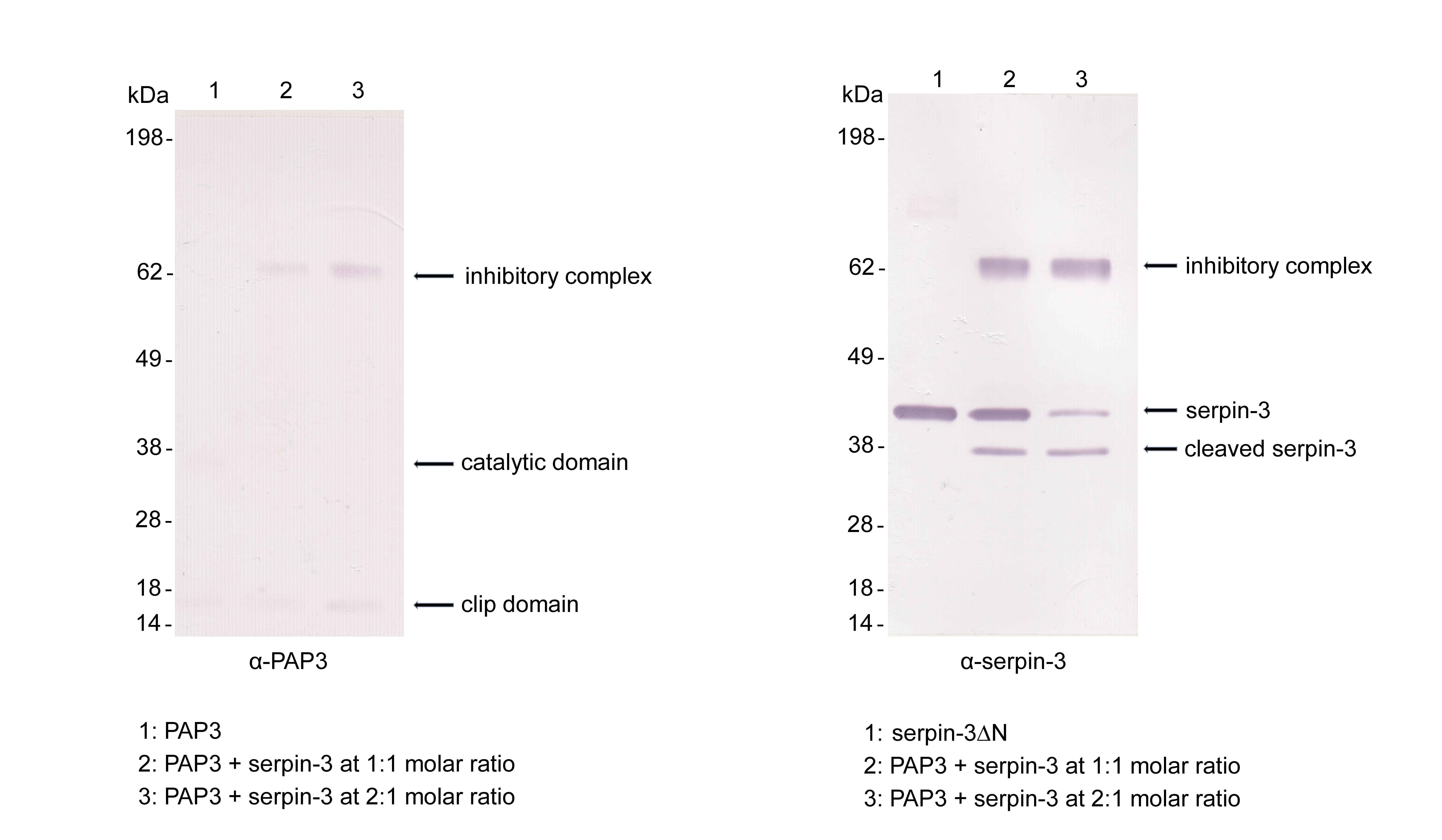


**Figure S3**: Interference of proPO activation in induced plasma by Ac-SVAFS-NH_2_ and Ac-SVAFS-COO^-^ treated serpin-3∆N (biological replicates). (**a**) Ac-SVAFS-COO^-^ treated serpin-3∆N (0.1 µg) or untreated serpin-3∆N (0.1 µg) was incubated with 2 µl plasma from *M. sexta* larvae at room temperature for 10 min, followed by addition of 2 µg *M. luteus* or sterile saline and further incubation for 10 min at room temperature. PO activities were measured by adding 2 mM dopamine. (**b**) 1 µl plasma from *M. sexta* larvae was incubated with pre-dried Ac-SVAFS-NH_2_ (5 nmol) or TFE in 50 mM sodium phosphate, pH 6.5 at room temperature for 1 h, followed by addition of 2 µg *M. luteus* or sterile saline and further incubation for 10 min at room temperature. PO activities were measured by adding 2 mM dopamine. Each graph represents results from individual plasma sample. One unit of proPO activity is defined as 0.001 change of OD_470_ per minute. Means ± standard deviation are from technical replicates (n=3). Results of statistical analysis (one-way ANOVA followed by Tukey’s multiple comparison test, P<0.05) are indicated with different letters.


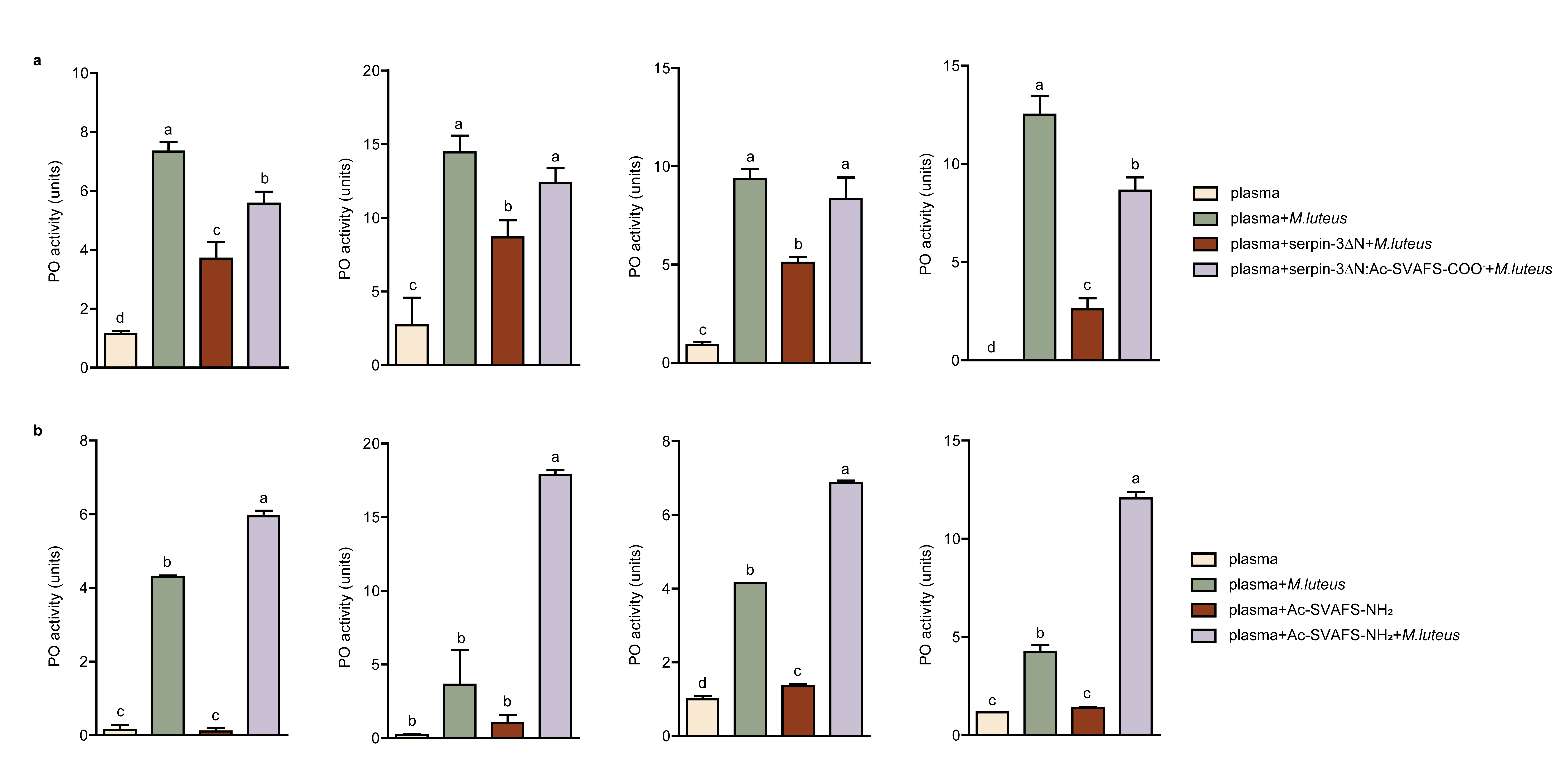

Supplement: Supplementary file 1 — Supplementary information [file 41598_2020_68316_MOESM1_ESM.docx]
